# Supplementary material for: Environmentally Friendly New Catalyst Using Waste Alkaline Solution from Aluminum Production for the Synthesis of Biodiesel in Aqueous Medium
Source: Bioengineering (Basel). 2023 Jun 7;10(6):692. doi: 10.3390/bioengineering10060692 (PMC10295760; doi:10.3390/bioengineering10060692)
Supplement: Supplementary file 1 [file bioengineering-10-00692-s001.zip › bioengineering-2408309-supplementary.pdf]

Supplementary material

Environmentally Friendly New Catalyst Using Waste Alkaline Solution from Aluminum Production for the Synthesis of Biodiesel in Aqueous Medium

Sandro L. Barbosa [1,*], David Lee Nelson [1], Lucas Paconio [1], Moises Pedro [1], Wallans Torres Pio dos Santos [1], Alexandre P. Wentz [1], Fernando L. P. Pessoa [2], Foster A. Agblevor [3], Daniel A. Bortoleto [4], Maria B. de Freitas-Marques [5] and Lucas D. Zanatta [6]

[1] Department of Pharmacy, Federal University of Jequitinhonha and Mucuri Valleys-UFVJM, Campus JK, Rodovia MGT 367–Km 583, nº 5.000, Alto da Jacuba, Diamantina 39100-000, Brazil; dleenelson@gmail.com (D.L.N.); lucas.paconio@ufvjm.edu.br (L.P.); moises.pedro@ufvjm.edu.br (M.P.); wallanst@ufvjm.edu.br (W.T.P.d.S.); wentzap@hotmail.com (A.P.W.)

[2] University center SENAI-CIMATEC, Av. Orlando Gomes, 1845, Piatã, Salvador 41650-010, Brazil; fernando.pessoa@fieb.org.br

[3] Utah Science Technology and Research (USTAR), Biological Engineering, Utah State University, Logan UT620 East 1600 North, Suite 130, Logan, UT 84341, USA; foster.agblevor@usu.edu

[4] Department of Geosciences, Universidade Federal do Pará, R. Augusto Corrêa, 01–Guamá, Belém 66075-110, Brazil; dabortoleto@yahoo.com.br

[5] Department of Chemistry, Instituto de Ciências Exatas, Universidade Federal de Minas Gerais. Av. Antônio Carlos, 6627, Pampulha, Belo Horizonte 31270-901, Brazil; betanialf@hotmail.com

[6] Laboratório de Química Bioinorgânica, Departamento de Química, Faculdade de Filosofia, Ciências e Letras de Ribeirão Preto, Universidade de São Paulo, Av. Bandeirantes, 3900, Ribeirão Preto 14040-901, Brazil; lucaszanatta@alumni.usp.br

* Correspondence: sandro.barbosa@ufvjm.edu.br; Tel.: +55-38-3532-1234

Abstract: Red mud (RM) is composed of a waste alkaline solution (pH = 13.3) obtained from the production of alumina. It contains high concentrations of soluble hematite (Fe_2O_3), goetite (FeOOH), gibisite [$Al(OH)_3$], a boemite (AlOOH), anatase (Tetragonal - TiO_2), rutile (Ditetragonal dipyramidal - TiO_2), hydrogarnets [$Ca_3Al_2(SiO_4)_{3-x}(OH)_{4x}$], and perovskite ($CaTiO_3$). It was shown to be an excellent catalytic mixture for biodiesel production. To demonstrate the value of RM, an environmentally friendly process of transesterification in aqueous medium using waste cooking oil (WCO), MeOH and waste alkaline solution (WAS) obtained from aluminum production was proposed. Triglycerides of WCO reacted with MeOH at 60 ºC to yield mixtures of fatty acid methyl esters (FAMEs) in the presence of 0.019% (w/w) WAS/WCO using the WAS (0.204 mol L^{-1}, predetermined by potentiometric titration) from aluminum production by the Bayer process. The use of the new catalyst (WAS) resulted in a high yield of the products (greater than 99% yield).

Keywords: Environmentally friendly processes; Bayer residue; waste management; basic catalyst, contaminants, red mud, fatty acid methyl ester.

Copies of spectra

1) WCO

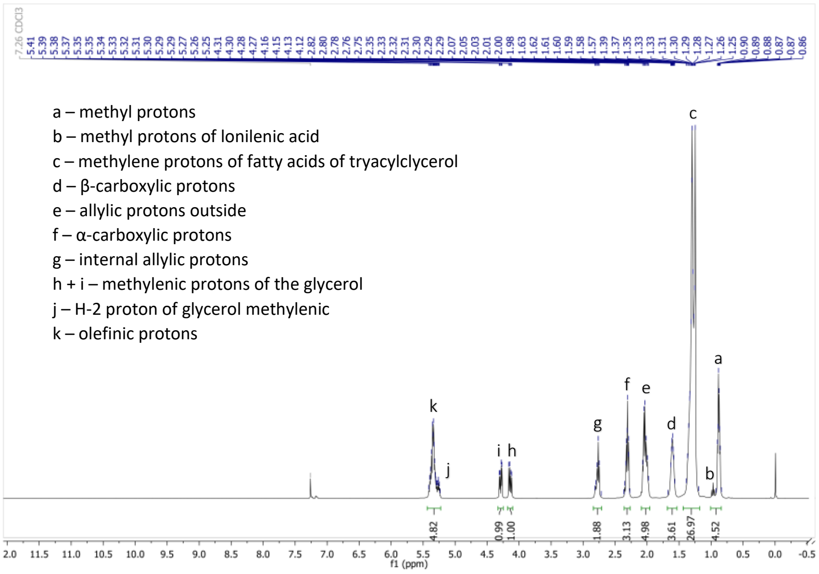

Figure S1. ^1H NMR WCO.

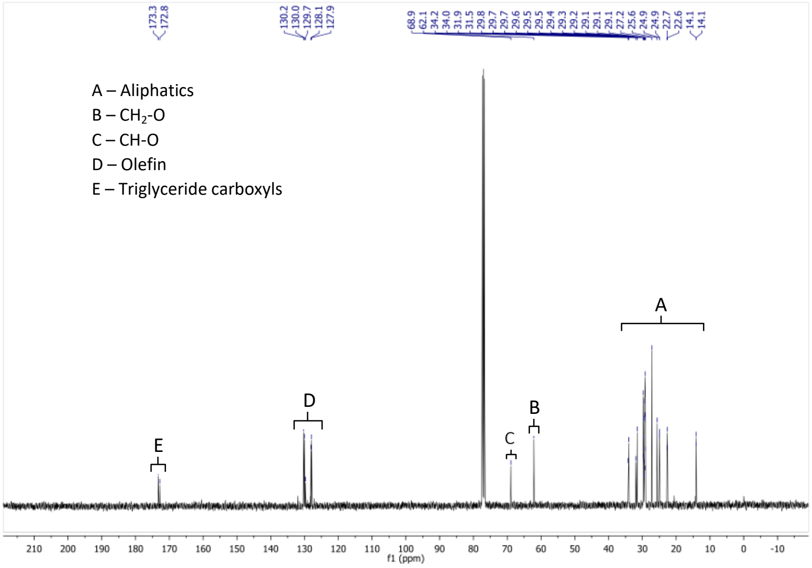

Figure S2. ^{13}C NMR WCO.

2) FAME (Fatty acid methyl ester)

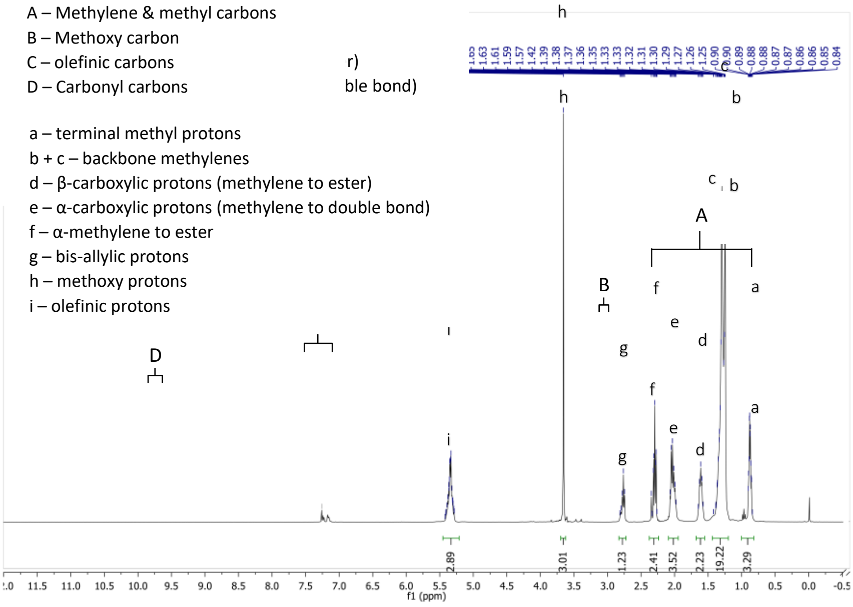

Figure S3. ^1H NMR FAME using WAS.

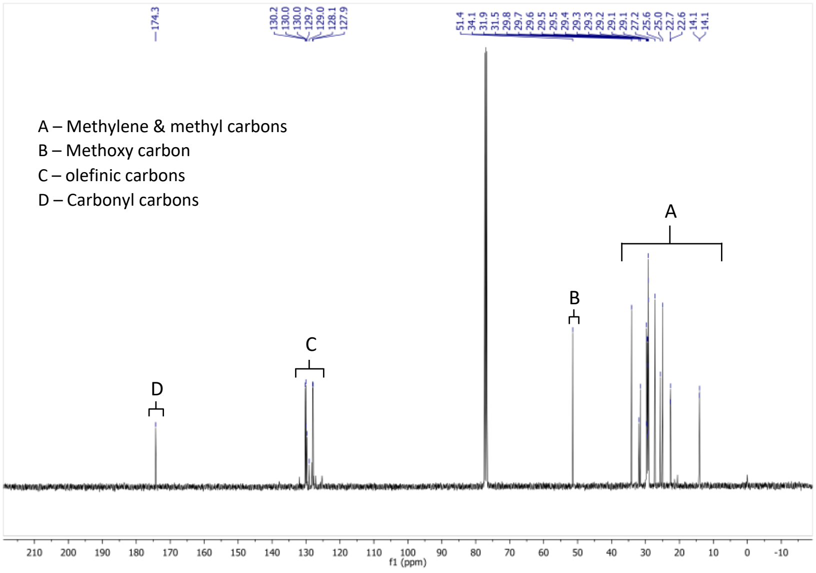

Figure S4. ^{13}C NMR FAME using WAS as catalyst.
